# Challenges in measuring measles case fatality ratios in settings without vital registration

**DOI:** 10.1186/1742-7622-7-4

**Published:** 2010-07-19

**Authors:** K Lisa Cairns, Robin Nandy, Rebecca F Grais

**Affiliations:** 1 Global Immunization Division, Centers for Disease Control and Prevention, 1600 Clifton Rd, MS E-05, Atlanta, GA 30333, USA; 2 Health Section, UNICEF, 3 UN Plaza, New York, NY 10017, USA; 3 Epicentre, 8 rue Saint Sabin, Paris, France 75011

## Abstract

Measles, a highly infectious vaccine-preventable viral disease, is potentially fatal. Historically, measles case-fatality ratios (CFRs) have been reported to vary from 0.1% in the developed world to as high as 30% in emergency settings. Estimates of the global burden of mortality from measles, critical to prioritizing measles vaccination among other health interventions, are highly sensitive to the CFR estimates used in modeling; however, due to the lack of reliable, up-to-date data, considerable debate exists as to what CFR estimates are appropriate to use. To determine current measles CFRs in high-burden settings without vital registration we have conducted six retrospective measles mortality studies in such settings. This paper examines the methodological challenges of this work and our solutions to these challenges, including the integration of lessons from retrospective all-cause mortality studies into CFR studies, approaches to laboratory confirmation of outbreaks, and means of obtaining a representative sample of case-patients. Our experiences are relevant to those conducting retrospective CFR studies for measles or other diseases, and to those interested in all-cause mortality studies.

## Introduction

Measles, a highly infectious vaccine-preventable viral disease, is characterized by clustering of cases that occur during cyclical epidemics [1]. In many parts of the world, measles is also a seasonal disease with fewer cases found during the non-measles season [2]. Clinically, the infection is expressed as a maculopapular rash accompanied by fever and at least one of the three "c's": cough, coryza and conjunctivitis; virtually all cases of measles are clinically expressed [3,4]. Measles is a potentially fatal disease [1]. The World Health Organization (WHO) defines a measles-associated death as one occurring within 30 days of rash onset, not obviously due to another cause such as trauma [5].

Historically, measles case fatality ratios (CFRs) have been reported to vary from 0.1% [1] in the developed world to as high as 30% among refugee populations [6,7]. Current estimates of CFRs used by WHO in endemic countries range between 0.05% - 6% [8-10]. Factors thought to affect CFR include age [11], intensity of exposure to measles virus (for which household crowding may be seen as a surrogate) [12], measles vaccination status [13], nutritional status [14], immunodeficiency [15] and access to appropriate case management [16]. Studies conducted in the late 1980 s demonstrated that supplementation of measles case-patients with vitamin A could decrease measles mortality by as much as 64% [17,18] leading to recommendations by WHO and United Nations Children's Fund (UNICEF) in 1987 to treat all measles case-patients with vitamin A in areas where measles CFRs were greater than 1% [19]. These recommendations, in conjunction with the rollout of Integrated Management of Childhood Illness (IMCI) guidelines in the mid 1990 s [20] which target pneumonia and diarrhea, might be anticipated to have decreased measles CFRs since the 1990 s. However, few data exist on the extent to which these interventions are used in health facilities, particularly in countries that are highly endemic for measles. Anecdotal evidence from outbreak investigations in Niger, Sudan and South Africa indicate that these interventions are underused [21-24].

Further decrease in CFRs in the past decade may have occurred due to the renewed political will and creation of the Measles Initiative in 2001. WHO and UNICEF developed a comprehensive strategy for sustainable measles mortality reduction with the goal of a 90% reduction in global measles deaths (compared with 2000 levels) by 2010.

The four-pronged strategy focuses on improved routine immunization, providing all children with a second dose of measles vaccine delivered either through periodic SIAs or routine services, improved measles case management and careful measles surveillance. This strategy has contributed to reducing the overall burden of measles and has potentially led to decreased CFR linked to earlier detection and improved case management.

Recently the global burden of mortality from measles, critical in prioritizing measles vaccination relative to other health interventions, has been an area of much discussion. A published point estimate from a 2003 analysis of childhood mortality using a proportional mortality model [25,26] differed by hundreds of thousands of deaths from the WHO point estimate for the same period using a static natural history model [27]. A major factor contributing to this discrepancy has been disagreement over which CFRs are appropriate to use in models seeking to estimate global burden of measles deaths, in particular what CFRs are appropriate to use in countries that are highly endemic for measles. In these settings, disease reporting, death surveillance and vital registration tend to be incomplete or non-existent, requiring the collection of primary data to determine disease-specific CFRs.

The recent controversy surrounding measles CFRs in the developing world and the paucity of up-to-date data on this topic have led us to conduct six published studies to determine measles CFR in five countries (Niger [21,24], Sudan [22], Nepal [28], Chad [24] and Nigeria [24], Table 1). Although a recent publication reviewed existing literature to better estimate probable CFRs by geographic region [10], there is little published guidance on the conduct of field studies to determine measles CFR. This paper aims to fill this gap by summarizing both the challenges that are inherent in such studies, and the approach that we advocate given these challenges. Improving the rigor of future studies in this topic area will contribute to more accurate burden of mortality estimates.

**Table 1:** Summary of published studies retrospectively estimating measles case fatality ratios and conducted by the authors

Reference	Location	Study type	Sampling	Recall Period	Study dates	Case Ascertainment	Lab Confirmation	Case Fatality Ratio
21	Mirrah district, Niger	Retro-spective	22 villages from 5 health districts selected based on number of cases and ability to lab confirm outbreak	Jan 1 - Apr 15 2003	May 24 - June 28 2003	House to house search; house-hold census in houses reporting cases; repeat visits to confirm deaths	5-10 serum samples in 12 of 22 villages surveyed	9.7% (95% CI: 7.9 - 11.5)

24	Boukoki, Niamey, Niger	Retro-spective community-based	One neighborhood selected based on population size and feasibility	Approx 6 months preceding survey	May 5 - 12, 2004	House to house search, collection of demographic and measles case data in all households	At least 10 cases lab-confirmed per epidemic	4.6% in children aged < 5 yrs

22	White Nile and Khartoum states, North Sudan	Retro-spective community-based	One administrative unit in each of two states selected on basis of number of measles cases; within each administrative unit, 10 villages randomly selected	Oct 1 2003 - Apr 30 2004	May 17 - July 8, 2004	House to house search for measles cases; house-hold census in houses reporting cases	Laboratory confirmation in each administrative unit	0.9% (95% CI: 0.16 - 1.91)

28	Nepal -- nationwide	Retro-spective community-based	Two stage random sampling for a total of one outbreak from each of 37 districts	March 1 - Sept 1 2004	Sept 2004 - Jan 2005	House to house search for measles cases; house-hold census in houses reporting cases	Laboratory confirmation of outbreaks	1.1% (95% CI: 0.5 - 2.3)

24	Moursal, Ndjamena, Chad	Retro-spective community-based	One neighborhood selected based on population size and feasibility	Approx 6 months preceding survey	July 4 - 12, 2005	House to house search, collection of demographic and measles case data in all household	At least 10 cases lab-confirmed per epidemic	4.0% in children aged < 5 yrs

24	Dong District, Nigeria	Retro-spective community-based	One neighborhood selected based on population size and feasibility	Approx 6 months preceding survey	Apr 25 - May 2, 2005	House to house search, collection of demographic and measles case data in all household	At least 10 cases lab-confirmed per epidemic	10.8% in children aged < 5 yrs

## Analysis

We analyzed our experience in conducting measles CFR studies and drew on the published literature to review critical considerations in designing CFR studies. Below, we first review the key components of a CFR study. Second, we summarize and provide recommendations for the conduct of future studies.

### Study type

Prospective disease and death surveillance can be used to determine disease-specific CFRs. However, because highly effective interventions now exist to prevent cases and deaths, it is ethically unacceptable to follow measles outbreaks without offering vaccination to affected communities and optimal case management, including vitamin A supplementation, to case-patients. Further, because this standard of care may not be routinely available, the CFR from such a prospective study may not reflect the true background rate. As a result, the true risk of death may best be studied retrospectively by conducting a cross-sectional survey to determine the number of measles cases that occurred in a defined area during a pre-determined period, and the number of associated deaths.

In conducting a retrospective study of this type, a critical issue is defining the recall period. Guides for the conduct of retrospective mortality surveys and their strengths and weakness have been described in the literature [29,20]. The recall period must be long enough such that sufficient cases and deaths occur for a precise CFR calculation, but on the other hand, the longer the recall period, the greater the likelihood of recall bias. Because measles is a disease found primarily in epidemics, identifying an adequate number of measles cases to determine measles CFR with some precision generally requires selecting the recall period to fall within the epidemic. Particularly in settings where measles is highly seasonal, this may restrict the period of interest to only several months. The survey must then be conducted toward the end of or very shortly after the epidemic season. Furthermore, determining measles CFRs retrospectively requires identification of all cases of measles within a household, as well as all deaths occurring in measles case-patients within 30 days of rash-onset. (The definition of a measles case is discussed in the forthcoming section). These requirements presuppose detailed recall, but permit focus on a short period (e.g., 3-6 months) prior to the survey.

### Ascertainment of cases and deaths

To accurately determine CFR, both the number of measles cases and associated deaths occurring during the recall period are needed. Literature on all-cause mortality shows that asking in aggregate about deaths occurring in the past 12-24 months results in 30%-40% under-reporting of deaths [30,31]. In addition, differential reporting by both age and gender [32] occurs, with the deaths most frequently omitted variously reported as those among young children [33] and those among children aged 5-15 years [30]. These findings on omission of childhood deaths have led to recommendations in the literature that mothers or other household women serve as respondents [34]. An additional difficulty concerns ascertaining the exact age of household members. Births and deaths are not routinely recorded and age is almost always imprecise. Further, rather than reporting deaths in aggregate, household enumeration should be conducted in order to permit every individual present in the household at the beginning of the recall period, or who joined the household during the recall period, to be accounted for [35,36]. We were unable to find literature on the accuracy of retrospective determination of measles cases. However, one might assume that steps recommended to minimize omission of deaths might also minimize the omission of cases.

A further challenge in conducting retrospective studies of measles is ensuring that the illness studied is indeed measles. Misclassification of rash-fever illness will result in inaccurate CFR estimates. Ensuring that the disease is measles is straightforward if cases have been laboratory confirmed. However, when this is not the case, it is necessary to rely upon history.

### Laboratory confirmation of measles cases

Although measles has distinctive clinical features, it can be mistaken for illness due to other causes, most frequently rubella. Thus, measles should be confirmed by collecting serum for testing between days 3 and 28 after rash onset [37]. The implementation of a global measles mortality strategy has led to improved surveillance for measles and the development of a global measles laboratory network [38,39], making laboratory confirmation of measles cases more widely available. However, development of laboratory capacity has been focused in countries that have initiated accelerated measles control, and, even in these settings, laboratories rapidly become overwhelmed during outbreaks [40]. As a result, current WHO recommendations are to limit laboratory confirmation to 5 - 10 specimens per outbreak, although the geographic area implicated (e.g., community, district) is undefined [41]. This recommendation takes into account the fact that the positive predictive value of the clinical case definition for measles increases greatly in the context of an outbreak [42].

The usual WHO laboratory protocol specifies testing for rubella if serum from a suspected measles case tests negative for measles. This has led to the recognition that some outbreaks thought clinically to be due to measles were in fact attributable to rubella, and has also led to confirmation of measles and rubella outbreaks occurring simultaneously in the same communities. In these circumstances, it is difficult to determine by history alone which rash-fever cases were due to measles and which to rubella, thus rendering accurate determination of disease-specific CFRs unfeasible.

In summary, in most measles-endemic settings it is rare that case-patients have laboratory confirmation of measles infection, although it is usually possible to test whether the rash-fever illness circulating is measles. Verifying measles infection in the actual case-patients that we wish to study - i.e., those that have survived 30 days after rash onset and those that have died - remains virtually impossible, as these are patients in whom it is too late to perform confirmatory laboratory testing.

### Use of verbal autopsy questions

As most measles cases in endemic settings are never subjected to laboratory testing, in retrospective studies history must be used to identify cases. Although a vast literature on verbal autopsies exists, these studies have focused primarily on use and validation of verbal autopsy methodology [43,44]. Verbal autopsy methodology has been extensively used in retrospective mortality studies addressing measles. Two approaches have been used: a clinical algorithm and the local term for measles. The algorithm using age ≥ 120 days, fever ≥ 3 days, and rash to identify measles cases has shown sensitivity ranging from 67% - 98%, and specificity ranging from 85% - 99% [45-47]. WHO recommends the use of either the algorithm or the local term [48]. In studies that compared this algorithm to the use of the local term for measles, the local term was more sensitive and more specific [49].

### Obtaining a representative sample of measles case-patients

In order to obtain a precise estimate of CFR, it is important to include an adequate number of measles cases in the sample. This number will depend upon the precision desired, as well as the pre-study estimate of CFR (Table 2). Estimates of probable CFRs for different regions have been published previously and can be used as a guide for determining sample size [10]. The level of precision desired will depend on the ultimate use of the CFR estimate, but should be as precise as possible. Ideally, the sample should be selected from a sampling frame of all case-patients resident in the geographic area of interest with rash onset during the period of interest. However, in the absence of a highly sensitive and specific surveillance system, such a sampling frame does not exist. Because measles tends to be highly clustered in both place and time, a sample drawn from the general population may result in a very large number of individuals being surveyed before an adequate number of measles case-patients is identified.

**Table 2:** Number of measles case-patients required to retrospectively estimate measles CFR based on expected CFR and desired precision

	95% Confidence Intervals					
**Expected CFR**	**± 0.5%**	**± 1%**	**± 2%**	**± 3%**	**± 4%**	**± 5%**

1%	1519					

2%	3003	752				

3%	4452	1117	279			

4%	5866	1473	369	164		

5%	7246	1821	456	203	114	

10%	13641	3445	864	384	216	138

15%	19215	4874	1223	544	306	196

20%	23995	6109	1534	682	384	246

Note: Sample sizes will need to be adjusted based on design effect and response rate

### Risk factors

In addition to estimating CFR, one would like to assess risk factors for increased or decreased CFR among the population studied.

#### Nutritional status

Poor nutritional status has been thought to be associated with increased measles CFR, and so one would like to know the nutritional status of measles case-patients at the time of rash onset. However, when conducting a retrospective study in a remote location, this information is rarely available. Instead, some have considered using the nutritional status of family members or of the general community at the time of the survey. However, this approach assumes that nutritional status of those still alive is representative of the nutritional status at disease onset of those who died - which appears unlikely. In some very poor rural communities dependent on subsistence agriculture, there is seasonal fluctuation in the prevalence of wasting. As a result, the community nutritional status during the CFR investigation may not reflect the community nutritional status a few months earlier during the measles outbreak. Logistically, determining current nutritional status adds complexity to field work as enumerators must be trained in anthropometry. If the nutritional status of the community as a whole is to be considered, a population-based sample must be selected for study.

#### Vaccination status

Children who received at least one dose of measles-containing vaccine have a lower CFR and reduced complications. Milder measles disease is also associated with a lower CFR in vaccinated children. In some contexts, proof of vaccination can be verified retrospectively from vaccination cards provided through routine vaccination systems or punctual mass vaccination interventions. In this case, the case-patients' vaccination status can be recorded. However, in many instances, card verification is not possible and history must be relied upon. This can be achieved by asking the mother if the child was vaccinated in the upper left arm for measles to avoid confusion with other antigens, but the possibility of response bias is always present. However, previous studies in areas of high measles incidence have shown parental recall to be highly reliable with a predictive value of approximately 95% [50].

#### Receipt of vitamin A during measles illness

Receipt of vitamin A during measles illness has been shown to result in a marked reduction in measles mortality. One would therefore also like to know if measles case-patients received vitamin A while ill and, if so, how many doses were received. Since vitamin A is generally given through health services, it may be possible to determine whether the patient received supplementation through record review. Alternatively, study respondents may be shown a vitamin A capsule and asked whether the case-patient received similar capsules. However, data on vitamin A supplementation during measles illness only support giving two age-appropriate doses of vitamin A separated by 24 hours, as a single dose has not been shown to reduce measles mortality [51]. Record documentation is rarely adequate to determine whether two doses were administered. Furthermore, in countries conducting frequent polio National Immunization Days (NIDs), respondents may confuse the drops of medication from a vitamin A capsule with the drops of oral polio vaccine. In summary, it is quite challenging to obtain accurate information on the receipt of vitamin A and, if received, the number of doses.

#### Case management

Although quality of case management may be an important risk factor for death, adequate evaluation is complex. The major clinical complications of measles leading to death are pneumonia and diarrhea. Evaluation of case management for these includes issues such as appropriate determination of pneumonia and diarrhea, and appropriate choice and dosing of antibiotics (for pneumonia) or appropriate use of oral rehydration solution (for diarrhea).

## Our Approach to Estimating Measles CFR

Taking into account the relevant literature, field experience, and the many challenges mentioned above, we have gradually developed a standardized approach to conducting measles CFR studies.

### Study type

We conducted only retrospective studies for the reasons mentioned previously. In order to best identify all cases and deaths, we used a household census approach, conducting a complete household census of those resident in the household at the beginning of the recall period and using this list to identify those who developed rash illness and died within thirty days of rash-onset. Whenever possible, we limited respondents to mothers or, if they were unavailable, other women within the household. Furthermore, we have limited the recall period to 3-12 months prior to the date of the survey, with the start and end of the period corresponding to a major local festival or event. All studies were conducted at the end of the epidemic season or shortly after.

In one study [21] we had the survey supervisors revisit households with reported measles deaths to confirm the numbers of measles cases and deaths. In addition, one of the primary investigators conducted follow-up household visits to ascertain whether the number of measles cases and deaths detected at the first visit matched those at the second visit, prioritizing households in which the questionnaire responses were unclear or incomplete. These follow-up visits comprised approximately 10% of households.

Despite increasing the likelihood that measles cases and deaths will be accurately detected, this approach has its own difficulties: household enumeration is time-consuming, and respondents may be suspicious of the motives for enumeration. This is particularly challenging when conducting studies in refugee or displaced populations, in which respondents may infer that benefits are tied to their response.

### Ascertainment of cases and deaths

To maximize the likelihood that the rash-fever illness we are studying is truly measles, we have opted to confirm virologically cases in the same community if the outbreak is still ongoing or, if necessary, in a neighboring community to which the community being studied has epidemiologic links. At times, we have also chosen to restrict the communities that we consider for study to those that have had laboratory confirmation of circulating measles [21,22,28]. We have also encountered co-circulating rubella and measles epidemics [28]. In these situations, because of the difficulty in differentiating measles and rubella on clinical grounds, a retrospective study to determine measles CFR may not be feasible.

We initially asked about measles infection using questions extracted from a standardized verbal autopsy questionnaire. However, when we used the algorithm [42-44], we doubted our results because they suggested isolated cases of measles in a poorly vaccinated population, a finding inconsistent with the epidemiology of the disease. We have since chosen to ask about measles infection by using the local term for the disease and then verifying that cases thus identified met the standard clinical case definition for measles, i.e., fever, rash and at least one of the following: cough, coryza and conjunctivitis, in case the term for measles in the local language covers other rash fever illnesses. We recommend this latter approach.

### Obtaining a representative sample

One of the greatest challenges has been identifying a method to select a representative sample of cases. Ultimately, we have chosen to select randomly communities (or other administrative units) from which several measles cases have been reported, and to conduct comprehensive, active, house-to-house case-searches within these communities. With this approach, all case-patients in communities eligible for study have an equal probability of selection, and findings, including the CFR, can be generalized to the population of case-patients in the communities studied. A confidence interval that accounts for clustering can be calculated. This approach may be criticized for failing to include sporadic cases reported to be measles. As indicated earlier, clustering of cases may lead to increased CFR through increased intensity of viral exposure; focusing solely on clustered cases could theoretically lead to an overestimate of true CFR. However, this is unlikely to have a major influence, because the vast majority of cases occur in outbreak settings and so even if sporadic cases were in fact true measles cases, they contribute relatively little to the total burden of disease. Furthermore, sporadic cases reported to be measles and meeting clinical criteria are far less likely to be true measles cases than clinically or laboratory-confirmed measles cases in laboratory-confirmed outbreak settings, because the positive predictive value of a case definition is much lower when the prevalence of measles is low [52].

### Risk factors

We have generally felt that the logistical difficulties of conducting a community-wide nutritional survey outweighed the potential benefits of doing so. We have chosen to rely instead upon data from the most recent Multiple Indicator Cluster Survey [53] or Demographic and Health Survey [54] to give an indication of probable community nutritional status, while understanding that these data may be neither geographically nor temporally specific to the outbreak under investigation. These surveys are internationally recognized, use standardized methodologies, permit cross-country comparisons, and frequently provide the only information available on country-level nutritional status. If there is no compelling reason to suspect rapid change in nutritional status, estimates from past surveys may give a rough idea of the importance of malnutrition in contributing to elevated CFRs.

We have tried to assess receipt of vitamin A by showing respondents a vitamin A capsule and asking whether the case-patient received similar medication. Although we have also asked about number of doses received, we have questioned respondents' ability to recall such detail, particularly several months after the fact. In one instance, receipt of vitamin A did not show any impact on measles mortality; leading us to question the reliability of the responses we received [21].

### Case management

Because of the complexity and additional length of including evaluation of measles case management in our surveys, and because our focus was primarily on determining CFRs rather than evaluating risk factors for death, we chose not to evaluate measles case management in our surveys.

### Other considerations

We ensured strong supervision of survey workers with a supervisor responsible for no more than two or three teams. Conducting complete enumeration of households as well as travelling to randomly-selected but distant locations to conduct studies may add to financial costs. However, we consider these approaches to be critical to the rigor of the studies. Studies using convenience sampling or only considering hospitalized cases may be less costly to conduct, but could lead to biased CFR estimates.

## Conclusion

In conclusion, despite the importance of measuring current measles CFRs in measles-endemic settings for prioritizing measles vaccination relative to other health interventions, the ethical imperative to ensure optimal measles case management limits the generalizability of results from prospective studies. Retrospective studies are in turn limited by recall bias, difficulty ensuring that rash and fever cases are truly attributable to measles, difficulty in assessing important risk factors for increased CFR, and challenges in obtaining a representative sample of measles case-patients. Despite these constraints, we believe that accurate measles CFRs can be obtained from meticulously conducted retrospective studies as we have outlined that take into account the unique characteristics of the disease. Table 3 provides summary guidance for good conduct of retrospective measles CFR studies. Guidelines for conduct of retrospective mortality surveys have been published previously [30,35] and well as guidelines to aid in interpretation [55]. Using these guidelines as a basis, in addition to those presented in Table 3, will help to improve the conduct of retrospective measles CFR studies.

**Table 3:** Key recommendations for retrospective measles CFR studies in countries without vital registration

Survey	Recommendation
Timing of survey	Toward end of epidemic or very shortly thereafter

Recall period	3-12 months with start and end coinciding with major local festival or event

Household	Household enumeration should be conducted using past census. Female head of household should be interviewed

Laboratory confirmation	Laboratory confirmation of circulating measles in the community under study

Case Ascertainment	Clinical algorithm and local term

Sampling	Exhaustive house-to house survey in selected communities with reported cases

Survey teams	Training on measles, survey methods, case ascertainment with supervisors responsible for no more than 2 to3 teams

## Competing interests

The authors declare that they have no competing interests.

## Authors' contributions

KLC drafted the manuscript. RN and RFG revised the manuscript critically for important intellectual content. All authors read and approved the final manuscript.
